# A universal design to realize a tunable perfect absorber from infrared to microwaves

**DOI:** 10.1038/srep32589

**Published:** 2016-09-07

**Authors:** Rafik Smaali, Fatima Omeis, Antoine Moreau, Thierry Taliercio, Emmanuel Centeno

**Affiliations:** 1 Clermont Université, Université Blaise Pascal, Institut Pascal, BP 10448, F-63000 Clermont-Ferrand, France; 2 CNRS, UMR 6602, Institut Pascal, F-63177 Aubière, France; 3 Universitè Montpellier, IES, UMR 5214, F-34000, Montpellier, France; 4 CNRS, IES, UMR 5214, F-34000, Montpellier, France

## Abstract

We propose a design for an universal absorber, characterized by a resonance frequency that can be tuned from visible to microwave frequencies independently of the choice of the metal and the dielectrics involved. An almost perfect absorption up to 99.8% is demonstrated at resonance for all polarization states of light and for a very wide angular aperture. These properties originate from a magnetic Fabry-Perot mode that is confined in a dielectric spacer of *λ*/100 thickness by a metamaterial layer and a mirror. An extraordinary large funneling through nano-slits explains how light can be trapped in the structure. Simple scaling laws can be used as a recipe to design ultra-thin perfect absorbers whatever the materials and the desired resonance wavelength, making our design truly universal.

The control of light absorbance plays a fundamental role in today’s photonics technologies with strong impacts for solar energy harvesting or for light emitting and sensing components^1^,^2^,^3^. Since according the Kirchhoff’s law, perfect absorbers and emitters are equivalent, significant efforts are pursued to realize compact artificial materials presenting an almost perfect absorption in a selective spectral range, for any polarization or incidence angle^4^,^5^,^6^. Whatever the approaches considered based either on critical coupling or impedance matching effects, the targeted operating frequency usually imposes the choice of the materials constituting the absorbers and also strongly constraints the design. These limitation originates from the intrinsic nature of Maxwell’s equations that are invariant under a change of scale solely for non-dispersive materials. However, realistic materials are always dispersive and simple scaling laws cannot be applied to tackle the variation of the absorption loss. For example, plasmonic absorbers have proven to be effective for visible and infrared radiations while metamaterials are preferably used from the terahertz to the microwaves^7^,^8^,^9^,^10^,^11^,^12^,^13^. Another approach consists in modifying the material property such as its plasma frequency according to the targeted operating frequency^14^,^15^,^16^,^17^. For those relying on the critical coupling condition, the minimum size of the resonators is always larger than *λ*/20 despite the strategies involved to reduce the effective wavelength of the mode responsible for the resonance^18^,^19^.

Here, we propose a resonant absorber which is universal since its optical properties are independent from the choice of the metals and dielectrics involved for its realization. The absorption frequency is demonstrated to be tuned from infrared to microwave frequencies by following simple scaling laws for universal absorber made of noble metal or highly doped semiconductors. In both cases, perfect absorption is reached for incident angles up to 30° and for any polarization of light. The universal absorber is in addition demonstrated to support a Fabry-Perot (FP) builds up in a near-zero dielectric thickness of *λ*/100 leading to an ultra-thin structure. This resonance is activated by a funneling effect through slits of few nanometers wide, with a ratio of the period to the width of the slits that can easily be larger than 30,000.

## Theory and design of super absorbers

**Figure 1:**
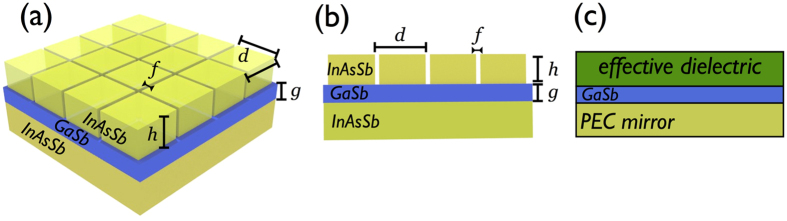
(**a**,**b**) Represent respectively the 2D and 1D metamaterial absorbers made of a grating of thin slits (width *f*) periodically etched (pitch *d*) in InAsSb, a GaSb spacer and a mirror. (**c**) Schematic of the equivalent systems consisting of an dielectric layer of effective index 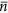 and thickness 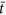, the GaSb spacer backed with a perfect electric conductor (PEC) mirror.

The absorber consists of a deeply subwavelength grating made of nanometers slits etched in a thin metallic slab which is separated from a metallic back mirror by a dielectric spacer, Fig. 1. The operating frequency range extends from the infrared to microwaves frequency when noble metals such as gold or silver are utilized (see Supplementary section A). The upper frequency boundary is limited by the plasma frequency of the metallic medium that is typically located in the ultraviolet spectrum for noble metals but can be located in the mid-infrared for highly doped semiconductors. Without any loss of generality, we illustrate our results by considering InAsSb, a highly doped semiconductor whose plasma frequency can be tuned by playing with the doping concentration. This material is in addition compatible with CMOS technology and its relative permittivity is given by a Drude model 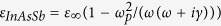 with 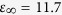, *ω*_*p*_ = 351.10^12^ *rad*.*s*^−1^ and *γ* = 10^13^ *rad*.*s*^−1^^14^,^20^. The spacer, of thickness *g*, is filled with a GaSb insulator (that is assumed to be non-dispersive) of refractive index *n*_*d*_ = 3.7. The absorbance *A* is deduced from the energy reflection coefficient *R* computed using the Rigorous Coupled-Wave Analysis (RCWA)^21^. First, we consider a 1D periodic set of slits of width *f* = 10 *nm* etched in the x-direction, with a pitch *d* = 2 *μm* and a thickness *h* = 320 *nm* for the metamaterial layer.

**Figure 2:**
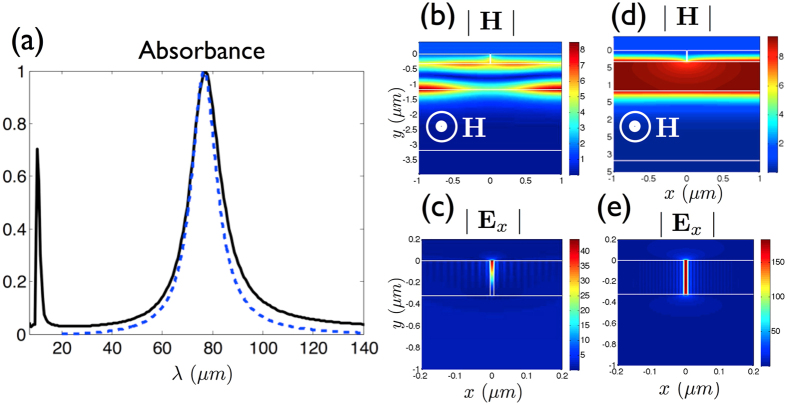
(**a**) Absorbance for a 1D metamaterial absorber (*f* = 10 *nm*, *d* = 2 *μm*, g = 850 nm). The solid and dashed curves are respectively obtained with the exact electromagnetic simulation and with the equivalent dielectric model. (**b**,**c**) Maps of the modulus of the magnetic and electric fields corresponding to *λ*_*s*_ = 11 *μm*. The p-polarized magnetic field is indicated on (**b**,**d**). (**d**,**e**) Maps of the modulus of the magnetic and electric fields corresponding to de FP resonance *λ*_*r*_ = 77 *μm*. Note that (**c**,**e**) are zoomed around the slit.

Two absorption lines can be observed in normal incidence on the computed absorption spectrum, for p-polarization case (*i.e.* a magnetic field along the slits in the z-direction), Fig. 2a. The lower resonant wavelength, *λ*_*s*_ = 11 *μm*, corresponds to a cavity-like mode localized into the slits, Fig. 2b,c. Similarly to the case of Extraordinary Optical Transmission (EOT)^22^, a gap-plasmon having a high effective index 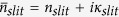 is excited and reflected at the top and at the bottom of the metamaterial layer. The real part of the effective index is well approximated by 
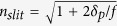
 where 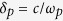 is the penetration depth into the metal^19^,^23^. The resonant condition for the slit reads 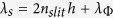 where 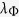 is a phase shift linked to the reflection coefficient of the gap-plasmon inside the metamaterial layer^24^,^25^. Remark that the spectral position of this slit mode is insensitive to an increase of the period. The spatial extension of the magnetic field for the second absorption line at *λ*_*r*_ = 77 *μm* indicates that it can be assimilated to a symmetric Fabry-Perot resonance localized inside the spacer of sub-wavelength thickness *g* = 850 *nm* (about *λ*/100) well below the common quarter-wavelength criterium, Fig. 2d,e.

We now derive an equivalent dielectric model to explain this actual reduction of the FP cavity size and to demonstrate that its excitation arises independently from the materials involved into the structure. Among the homogenization technics used for plasmonics systems, it could be attractive to replace the grating by a layer whose effective permittivity tensor can be deduced from Maxwell-Garnett theory. This approach can be applied very effectively to metallo-dielectric multilayers^26^,^27^,^28^,^29^ or randomly distributed nanoparticles^30^. However, when the gap plasmon resonance dictates the optical response of the structure, it is necessary to use the approach of ref. 31 that makes the equivalence of a metallic grating to an effective dielectric slab of complex refractive index 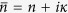 and thickness 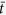, Fig. 1c. The optical property of such an artificial dielectric layer is known to depend on the geometrical parameters of the grating and on the effective index of the gap plasmon by 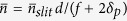
25. We underline that for very narrow slits, we obtain a dielectric layer of very high effective index 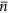 whereas Maxwell-Garnett formulas predict instead an almost metallic effective layer. The resonant wavelength of the gap plasmon mode is equivalently linked to the effective index and thickness by 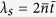. With these definitions, the whole 1D absorber can be replaced by a much simpler equivalent system made of a dielectric spacer sandwiched between a back mirror assumed to be a perfect electric conductor (PEC) and an absorbing layer of complex index 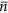 corresponding to the grating layer, Fig. 1c. As seen on Fig. 2a, this equivalent dielectric system reproduces the FP absorption line centered at *λ*_*r*_ = 77 *μm*. From this simpler structure, we search analytical expressions for realizing the resonant conditions of the FP mode. By taking into account the PEC back mirror, the magnetic field inside the spacer reads *H*_*z*_ = *B cos*(*k*_0_*n*_*d*_*y*), with *k*_0_ = 2*π*/*λ*. The electromagnetic continuity conditions applied at the interfaces lead to link by a **T** matrix the amplitude *B* of the spacer mode to the amplitudes *I* and *R* of the incident and reflected waves:


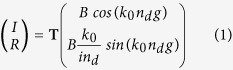


from which the amplitude of the FP mode is expressed in a conventional formulation:


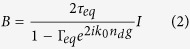


Here 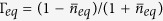 is an equivalent reflection Fresnel coefficient determined by the equivalent index 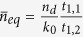 related to the elements *t*_*i*,*j*_ of the T-matrix. Remark that 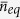 simply reduces to *n*_*d*_ when the grating is removed leading for Eq. (2) to the case of a single dielectric slab on top of a PEC mirror. The analytical expressions for *t*_1,2_ and *t*_1,1_ allows to write the equivalent index as


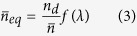


where 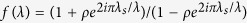 and 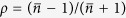 designates the Fresnel coefficient at the air-dielectric interface for p-polarized light. For thin slits (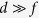) 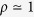 since 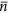 takes very high values. Thus, in the long wavelength limit, when 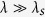, the function *f*(*λ*) can be approximated by 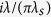. These simplifications leads to write the equivalent index in the following form:


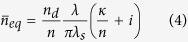


**Figure 3:**
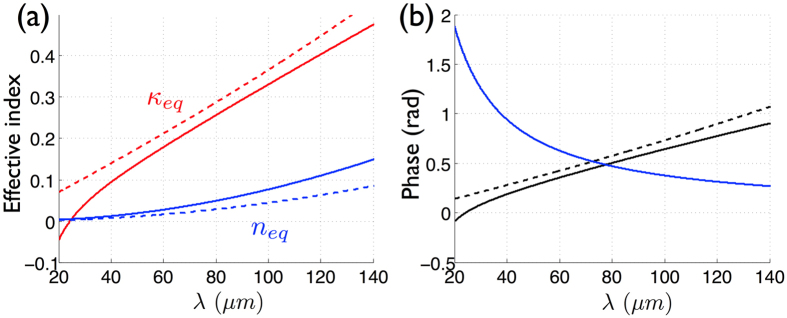
(**a**) Equivalent refractive index and extinction coefficient as a function of the wavelength obtained with Eq. (3) in solid curves and with the approximate formulation Eq. (4). (**b**) Phase terms Eq. (7): spacer phase 2*k*_*d*_*g* (blue curve), −arg(Γ_*eq*_) (black curve) and 2*κ*_*eq*_ (dashed curve).

Introducing the figure of merit (FOM) 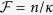, the equivalent extinction coefficient 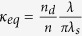 is inversely linked to the equivalent refractive index by 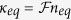. The good agreement between the exact expression of the complex equivalent index of Eq. (3) and that of the analytical one of Eq. (4) is shown in Fig. 3a. Equipped with this complex equivalent index, two optical conditions (one for phase and a second for the modulus) can be extracted from the magnetic FP resonance condition:


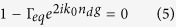


The first condition, 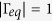, is satisfied for the trivial solution *n*_*eq*_ = 0 whatever the value of the equivalent absorption *κ*_*eq*_. In practice, almost perfect absorption higher than 98% is achieved when a near-zero equivalent index condition is satisfied, which is the case when *n*_*eq*_ = 0.1 for instance, see Fig. 3a. From the definition of *n*_*eq*_, we directly derive an analytical expression for the resonant wavelength:


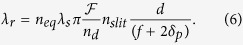


By considering the phase condition which implies that the FP mode is built up inside the spacer when the total phase is cancelled out, we obtain:


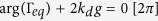


The first term of Eq. (7) can be written in terms of the equivalent refractive index and extinction coefficient as 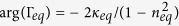. As seen on Fig. 3b, it is well approximated by arg (Γ_*eff*_) = −2*κ*_*eq*_ when the near-zero equivalent index condition is satisfied. The ratio *η* = *g*/*λ*_*r*_ of the spacer’s thickness over the wavelength thus given by the following equation:


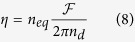


In order to get an absorbance higher than 95%, we have to set *n*_*eq*_ = 0.1. Hence, Eq. (8) shows that the resonant phase condition is driven by the figure of merit which is related to the properties of the gap plasmon mode into the slits : 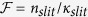. One could claim that 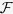 thus depends on the choice of the metal utilized for the grating layer. However, we have found that in the long wavelength limit 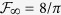 for any metals (noble or highly doped semiconductors) described by the Drude model (see Supplementary section B). This result demonstrate that our absorber is universal in the sense that its absorption mechanism does not depend from the metal choice and consequently is not affected by the metal dispersion. With these parameters we arrive to *η*  = 1.1/100, demonstrating that the FP resonance is better excited when the dielectric layer playing the role of a cavity has a thickness that is roughly only one hundredth of a wavelength. This property originates from the negative phase, −2*κ*_*eq*_, acquire by the electromagnetic waves when they are reflected by the equivalent high index dielectric layer. It turns out the RCWA allows to access this phase rigorously, by retrieving the actual reflection coefficient on the metamaterial layer (see Supplementary section C), and that such a computation totally confirms the analytical results. Inserting this phase condition into Eq. (6) and for nanometer slits (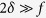), we get a simple expression for the resonant wavelength of the FP mode:


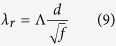


with 
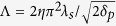
 that can be evaluated for 10-nanometer slits to Λ = 3.38. Finally, Eqs (8) and (9) provide simple scaling laws for designing universal absorbers operating at arbitrary large wavelengths.

## Perfect absorbers from infrared to microwave

**Figure 4:**
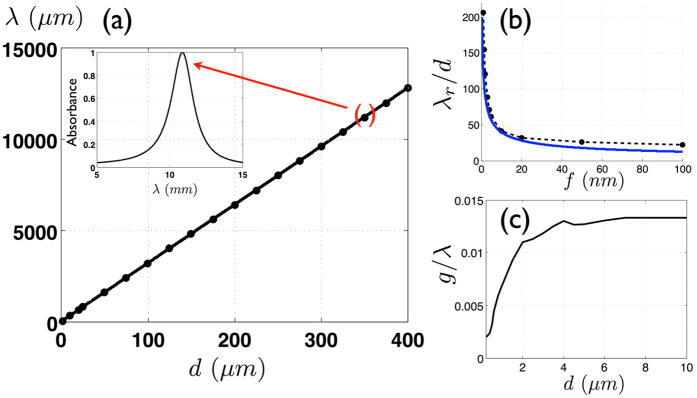
(**a**) Resonant wavelength *λ*_*r*_ as a function to the pitch *d* for a 1D absorber. The bold line corresponds to the theoretical scaling law *λ*_*r*_ = 33 *d* and the dots to the exact electromagnetic computations. The inset represents the absorbance spectrum with a total absorption in the microwave range for *λ*_*r*_ = 11.2 *mm* when *d* = 350 *μm*. (**b**) Ratio *λ*_*r*_/*d* with respect to the slit width, the dashed and solid curves are respectively obtained with the RCWA simulations and with Eq. (9) for *d* = 1 *μm*. (**c**) Ratio of the spacer over the wavelength with respect to the pitch. *g*/*λ* remains constant for a period larger than 8 *μm*.

These theoretical results are confirmed by the exact electromagnetic calculations of the absorbance *A* deduced from the reflectivity and performed in normal incidence with the RCWA method where 100 Fourier modes are used for the largest pitches. On Fig. 4a, the resonant wavelengths of the FP resonance are shown for grating periods *d* ranging from 1 *μm* to 400 *μm*. In agreement with our model, the resonant wavelength is seen to be linearly linked to the period by 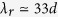 (for *d* in microns). The use of Eq. (9) for slits of 10 *nm* wide leads to a slope of 33.8, thus confirming the excellent accuracy of our analytical model. This means that the structure is able to absorb microwaves with a wavelength that is more than 6 orders of magnitude larger than the slits’ width (see Fig. 4a). The electric field associated to this FP magnetic resonance is actually squeezed in slits that are a million times smaller than the wavelength. This is where the absorption takes place, since the dielectric spacer is considered lossless. As seen on Fig. 4b, the dimension of the slits have an important impact on the spectral position of the magnetic FP resonance. This is especially true when they are a few nanometers wide, as this dimension has a very large influence on the effective index of the gap-plasmon propagating in the slits. A quite good agreement is observed with the exact results obtained using the RCWA simulations and Eq. (9) for *d* = 1 *μm*. Beyond a pitch of 4 *μm* or equivalently for wavelengths larger than 100 *μm*, the thickness of the spacer remains constant about *g*/*λ* = 1.3/100, close to the theoretical limit *η* = 1.1/100, Fig. 4c. For arbitrarily large wavelengths, the slits operate as antennas that funnel the incident waves into the spacer that constitutes the resonant cavity. The funneling factor, the ratio of the pitch to the slid width, is huge: it can be as large as 40,000 which is way above what usually happens for EOT when the resonance is located in the slits. This mechanism holds from the infrared to the microwave range despite the dispersive behavior of InAsSb and can be obtained for other metals such as silver (see Supplementary Fig. S1).

**Figure 5:**
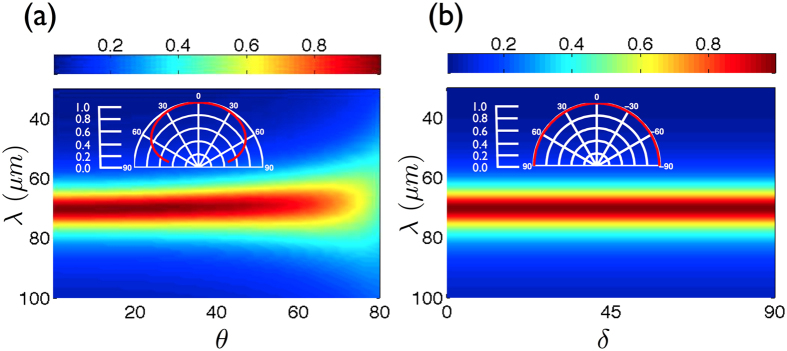
(**a**) Absorbance with respect to the incident angle *θ* and for a polarization angle *δ* = 0°. The inset represents the polar plot of the absorbance computed for the absorption line *λ*_*r*_ = 70 *μm*. (**b**) Absorbance with respect to the polarization angle *δ* and for normal incidence (TM and TE polarizations cases are respectively defined by *δ* = 0° and *δ* = 90°. In the inset shows the polar plot of the absorption line as a function of *δ*.

From the application point of view, realizing absorbers insensitive to the incident angle and to the polarization of light is a crucial issue. We address these problems by considering 2D metamaterial absorbers made of a square array of thin slits (width *f* = 10 *nm*) separated by a pitch *d* = 2 *μm*, Fig. 1a. We illustrate these properties for a targeted absorption line at *λ*_*r*_ = 70 *μm* leading to a spacer’s thickness *g* = 850 *nm*, a pitch *d* = 2 *μm* and a grating’s thickness *h*_*r*_ = 320 *nm*. More than 90% of the incident radiation is absorbed by the metamaterial for incident angles up to 50° and the absorbance reaches 70% at grazing incidence for 70°, Fig. 5a. The efficiency of the absorber is also seen to be insensitive to the polarization of light: the structure can simply be seen as two crossed gratings, each one being responsive to one polarization only. In normal incidence, the absorbance thus remains constant whatever the polarization in normal incidence.

## Conclusion

We have proposed a metamaterial resonant absorber whose absorption line can be chosen in any frequency range, from optics to microwaves, by following simple scaling laws, essentially. Our approach allows to design a perfect absorber that working for any frequency, independently of the materials that are considered. Almost perfect absorption can be obtained whatever the polarization over a broad incident angle range. This is why we think our design can be said to be *universal*. The metamaterial layer controlling the response of the structure allows to reduce to *λ*/100 the thickness of the spacing layer constituting a resonant cavity on which the device is based. The absorption takes place in slits that are no more than a few nanometers wide, despite wavelengths that are 1,000,000 times larger. All the incoming radiation in funneled through these slits despite a ratio of 1 to 40,000 between the slit width and the period. We have derived an analytical model thoroughly describing the electromagnetic response of the device that proved very accurate despite these extreme and unprecedented ratios. Universal absorbers constitute ultra-thin and flexible solutions for absorbing any kind of electromagnetic radiation especially at terahertz frequencies where absorbers, sensors and emitters are usually difficult to design. Integrating electric contacts into the device or making it dynamically tunable is something that can totally be envisaged, broadening even more the potential of our design.

## Additional Information

**How to cite this article**: Smaali, R. *et al*. A universal design to realize a tunable perfect absorber from infrared to microwaves. *Sci. Rep.* **6**, 32589; doi: 10.1038/srep32589 (2016).

## Supplementary Material

Supplementary Information
