# Supplementary material for: A universal design to realize a tunable perfect absorber from infrared to microwaves
Source: Sci Rep. 2016 Sep 7;6:32589. doi: 10.1038/srep32589 (PMC5013470; doi:10.1038/srep32589)
Supplement: Supplementary Information [file srep32589-s1.pdf]

A universal design to realize a tunable perfect absorber from infrared to microwaves: Supplementary information

Rafik Smaali, Fatima Omeis, Antoine Moreau, Thierry Taliercio, Emmanuel Centeno

ABSTRACT

This document provides supplementary information to "A universal design to realize a tunable perfect absorber from infrared to microwaves".

A. Universal absorber made of silver

In this section we demonstrate that universal perfect absorber can be realized when silver is used for the metallic layers. We consider a spacer with a refractive index $n_d = 3.7$, slits of width $f = 10\ nm$ etched in a grating whose thickness is $h = 20\ nm$. The slits support a gap plasmon resonance for $\lambda_s = 1\ \mu m$ which leads to a constant $\Lambda = 1.24$ (for a skin depth $\delta_p = 25\ nm$). The equivalent dielectric model leads to $g/\lambda_r = 1.1/100$ and $\lambda_r = 12.4d$ (for the pitch d expressed in microns). These results are confirmed by the direct computation of the absorption line corresponding to the magnetic FP resonance for different lattice periods, Fig. S1. We found a linear variation $\lambda_r = 14.2d$ in agreement with the theoretical prediction (with an error of 13%). The actual relative thickness of the spacer converges to 1.4/100, a value very close to the theoretical one, Fig. S1.b.

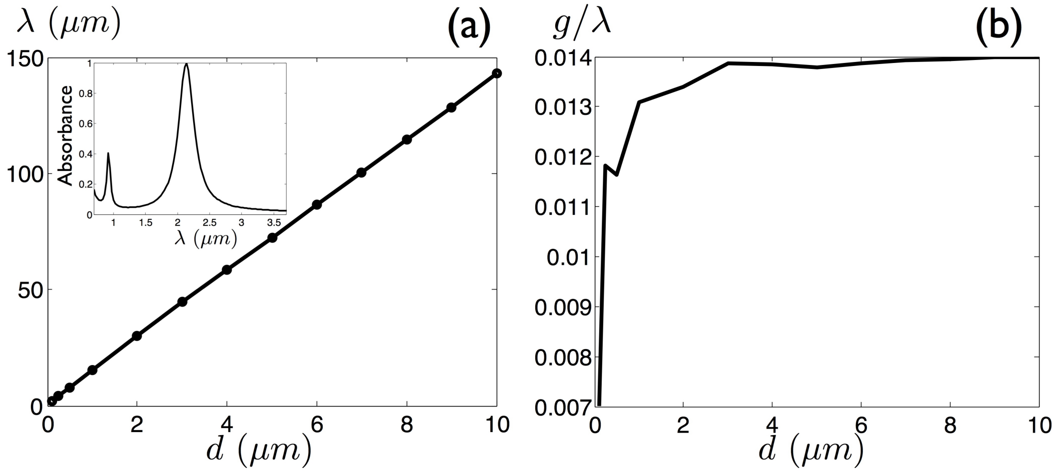

Figure S 1. (a) Resonant wavelength with respect to the pitch d for a silver 1D universal absorber with slits of width $f = 20\ nm$. (b) g/λ_r as a function of d.

B. Asymptotic formulation of the figure of merit

We demonstrate that in the quasi-static limit the figure of merit (FOM), $\mathscr{F} = n/\kappa$, reaches a limit which is independent from the metal choice. For that purpose, we consider a generalized Drude model for the relative permittivity of the metallic layers that be noble metals or highly doped semiconductors:

$$\bar{\varepsilon}_r = \varepsilon_\infty - \frac{\omega_p^2}{\omega^2 + i\gamma\omega} \tag{1}$$

In the long wavelength limit $\omega/\gamma \ll 1$ and the complex permittivity reads:

$$\bar{\varepsilon}_r = \varepsilon_\infty - \frac{\omega_p^2}{\gamma^2} + i\frac{\omega_p^2}{\gamma\omega} \tag{2}$$

In the case of noble metals $\varepsilon_\infty = 1$ and takes values about 10 for semiconductors. Hence (2) is well approximated by:

$$\bar{\varepsilon}_r = \frac{\omega_p^2}{\gamma\omega}(-\frac{\omega}{\gamma}+i) \tag{3}$$

The FOM is equivalently given by $\mathscr{F} = n/\kappa$ or $\mathscr{F} = n_s/\kappa_s$ because of the relationship of the equivalent refractive index $\bar{n}$ and the effective index of the gap plasmon $\bar{n}_{slit}$. For nanometer slits $\bar{n}_{slit} = \sqrt{\lambda/(\pi f\sqrt{-\varepsilon_r})}$[1,2]. By using (3), we get:

$$\bar{n}_{slit} = \sqrt{\frac{\lambda}{\pi f}}\left(\frac{\gamma\omega}{\omega_p^2}\right)^{1/4}\left(1-i\frac{\omega}{4\gamma}\right)e^{i\pi/8} \tag{4}$$

We write this complex index as:

$$\bar{n}_{slit} = \sqrt{\frac{\lambda}{\pi f}}\left(\frac{\gamma\omega}{\omega_p^2}\right)^{1/4}\left|1-i\frac{\omega}{4\gamma}\right|e^{i\theta_s} \tag{5}$$

where $\theta_s \simeq \pi/8 - \omega/(4\gamma)$. The FOM is thus given by $\mathscr{F} = 1/\tan\theta_s$ which is well approximated by:

$$\mathscr{F} = \frac{8}{\pi}(1+\frac{2\omega}{\pi\gamma}) \tag{6}$$

In the quasi-static limit, $\omega \to 0$, we finally get $\mathscr{F}_\infty = 8/\pi$ or $\mathscr{F}_\infty = 2.54$. Figure S2 represents the FOM with respect to the

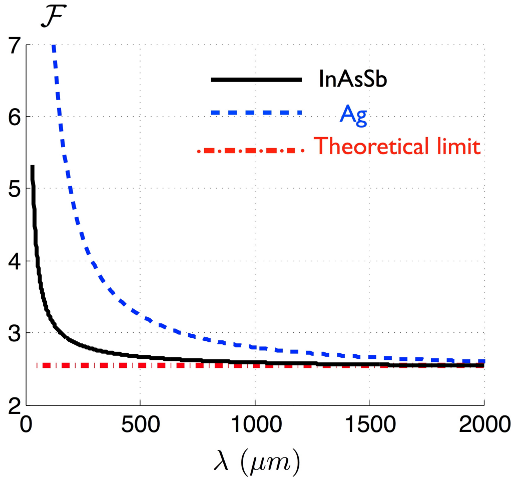

Figure S 2. Figure of merit with respect to the wavelength for a highly doped semiconductor (InAsSb in solid curve), a noble metal (Ag in dashed curve). The red dot curve shows the quasi-static limit $\mathscr{F}_\infty$.

wavelength for InAsbs and for silver[3]. It is seen that these FOMs reach $\mathscr{F}_\infty$ at large wavelengths. These results demonstrate that the FOM and thus that the phase condition for the universal absorbers holds independently from the choice of the metal.

C. Comparison of the exact and analytical phases for the equivalent Fresnel coefficient

In this section, we compare the phase of the equivalent Fresnel coefficient, $\arg(\Gamma_{eq})$, derived from the equivalent dielectric model and from the exact reflexion coefficient extracted from the RCWA code. The parameter for the 1D universal absorber are $f = 10$ nm, $d = 2$ μm, $g = 850$ nm, $h = 320$ nm. This phase was found to be negative around the absorption line and reaches the asymptotic function $-2\kappa_{eq}$, see dashed curve on Fig. S3.a. This is confirmed by the RCWA, a method which relies on the computation of the scattering matrices of sub-structures of the device. Scattering matrices explicitly contain the reflection and transmission coefficient related to any given sub-structure. It thus gives access to the reflection coefficient of a plane wave propagating inside the spacer in normal incidence on the metamaterial layer r_{MM}, and on the mirror r_{Mirror}. We have then

$$\arg(\Gamma_{RCWA}) = \arg(r_{MM}) + \arg(r_{Mirror}) \tag{7}$$

We derive the analytical expression of the total phase that cancels at the FP resonance, Fig. S3.b:

$$\Phi_{eq} = -2\kappa_{eq} + 2k_d g \quad (8)$$

and the exact total phase calculated from the RCWA method:

$$\Phi_{RCWA} = \arg(\Gamma_{RCWA}) + 2k_d\, g \quad (9)$$

where $k_d = 2\pi/\lambda n_d$. these results demonstrate that the metamaterial interface introduces a negative phase shift responsible for the near-zero thickness of the dielectric spacer.

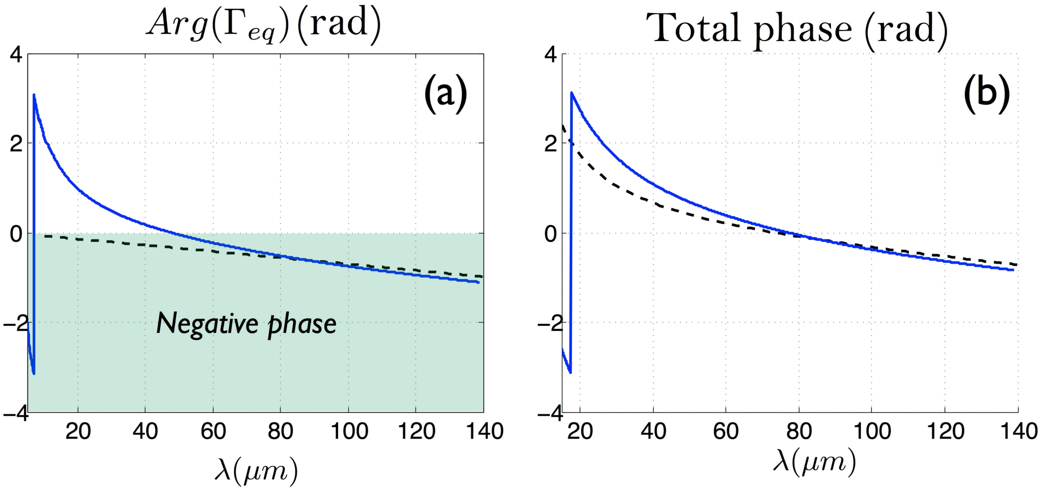

Figure S 3. (a) Phase of the equivalent Fresnel coefficient. The solid and dashed curves are respectively $\arg(\Gamma_{RCWA})$ and $-2\kappa_{eq}$. (b) Total phase through the metamaterial. The solid and dashed curves are respectively Φ_{RCWA} and Φ_{eq}.

References

1. Pardo, F., Bouchon, P., Haidar, R., Pelouard, J.-L. Light Funneling Mechanism Explained by Magnetoelectric Interference. *Phys. Rev. Lett.* **107**, 093902 (2011).
2. Collin, S., Pardo, F., Pelouard, J.-L. Waveguiding in nanoscale metallic apertures. *Opt. Express* **15**, 4310–4320 (2007).
3. Palik, E.D. "Handbook of Optical constants of solids," *Academic Press Inc.*, New York **1991**.
